# LRP-1-dependent control of calpain expression and activity: A new mechanism regulating thyroid carcinoma cell adhesion

**DOI:** 10.3389/fonc.2022.981927

**Published:** 2022-08-16

**Authors:** Benoit Langlois, Julie Martin, Christophe Schneider, Cathy Hachet, Christine Terryn, Damien Rioult, Laurent Martiny, Louis Théret, Stéphanie Salesse, Stéphane Dedieu

**Affiliations:** ^1^ UFR Sciences Exactes et Naturelles, Université de Reims Champagne-Ardenne, Reims, France; ^2^ Matrice Extracellulaire et Dynamique Cellulaire, MEDyC, UMR 7369 CNRS, Reims, France; ^3^ Plate-Forme Imagerie Cellulaire et Tissulaire (PICT), Université de Reims Champagne-Ardenne, UFR Médecine, Reims, France; ^4^ Plateau Technique Mobile de Cytométrie Environnementale MOBICYTE, Université de Reims Champagne-Ardenne/INERIS, Reims, France

**Keywords:** LRP-1, cancer, adhesion receptors, cell-matrix interactions, invasive potential

## Abstract

The low-density lipoprotein receptor-related protein 1 (LRP1) is a multifunctional endocytic receptor mediating the clearance of various molecules from the extracellular matrix. LRP1 also regulates cell surface expression of matrix receptors by modulating both extracellular and intracellular signals, though current knowledge of the underlying mechanisms remains partial in the frame of cancer cells interaction with matricellular substrates. In this study we identified that LRP1 downregulates calpain activity and calpain 2 transcriptional expression in an invasive thyroid carcinoma cell model. LRP1-dependent alleviation of calpain activity limits cell-matrix attachment strength and contributes to FTC133 cells invasive abilities in a modified Boyden chamber assays. In addition, using enzymatic assays and co-immunoprecipitation experiments, we demonstrated that LRP1 exerts post-translational inhibition of calpain activity through PKA-dependent phosphorylation of calpain-2. This LRP-1 dual mode of control of calpain activity fine-tunes carcinoma cell spreading. We showed that LRP1-mediated calpain inhibition participates in talin-positive focal adhesions dissolution and limits β1-integrin expression at carcinoma cell surface. In conclusion, we identified an additional and innovative intracellular mechanism which demonstrates LRP-1 pro-motile action in thyroid cancer cells. LRP-1 ability to specifically control calpain-2 expression and activity highlights a novel facet of its de-adhesion receptor status.

## Introduction

The Low-density lipoprotein (LDL) receptor-related protein-1 (LRP-1) is a large ubiquitously expressed multifunctional receptor, member of the LDL-receptor family (1). Being first identified as an endocytic receptor for α-2 macroglobulin (2, 3), LRP-1 demarks from the smaller isoforms of this ancient gene family by its ability to mediate the specific internalization and lysosomal targeting of over 30 distinct extracellular ligands. This includes proteases, protease-inhibitor complexes, matricellular proteins and growth factors (4). Initially synthesized as a 600 kDa precursor, LRP-1 is processed in the trans-Golgi by a furin-convertase to be addressed at cell surface in the mature two-chain form. It is composed of a 515 kDa extracellular α-chain, involved in the specific recognition of cell surface and soluble proteins, and a 85 kDa β-chain encompassing the transmembrane domain and a short cytoplasmic tail providing dual function to LRP-1 in the recruitment of adaptors of the endocytic machinery or signaling scaffolds (4, 5).

The diversity of cell surface interactions engaged by LRP-1 has attracted research interest in several pathological contexts including atherosclerosis, neurodegenerative and fibrotic disorders (6–8). Additionally, LRP-1 well documented function in the endocytic clearance of matrix proteases-containing complexes, including matrix metalloproteases (MMPs) (4), and plasminogen activators uPA and tPA (9–11), widely involved in invasive behavior of cancer cells, early pointed its crucial role in matrix remodeling events and designated its putative status of tumor suppressor (4, 12). Consistently, a weak expression of the receptor was observed in high grade cancer cells and tissues of various origins (11, 13, 14). However, this attractive model was counterbalanced by studies reporting LRP-1 positive contribution towards tumorigenesis and metastatic dissemination (15, 16). Moreover, the LRP-1 endocytic control of matrix proteolysis may be antagonized by its ability to control expression of matrix components (17) and proteases (18, 19) at the transcription level.

LRP-1 overall function in carcinogenesis therefore appears to be much more complex than initially assumed. Beyond endocytosis, LRP-1 was indeed reported to control activation of several intracellular signaling pathways by recruiting various scaffolding proteins on the two NPxY motifs of its intracellular domain (20, 21). In this way and in response to extracellular stimuli, LRP-1 may assemble specific signaling platforms to regulate cell proliferation, survival, differentiation and migration (22–25). Accordingly, LRP-1 dependent activation of the JAK/Stat pathway was involved in the pro-motile effect of the plasminogen activator inhibitor PAI-1 (26). In fibrosarcoma cells, LRP-1 deficiency appeared to limit invasive process by down-regulating the extracellular signal-regulated kinase (ERK) pathway (27, 28). In contrast, LRP-1 was shown to activate promigratory signals in various cell types (25, 29–31), including tumor cells (15, 19, 32–34). For instance, in breast (33) and pancreatic (34) cancer models, the secretion of HSP90α in the tumor microenvironment and its specific binding to LRP-1 at the tumor cell surface was recently linked to the induction of common, but also distinct features, of an epithelial-to-mesenchymal transition in favor of metastatic dissemination and correlated to poor prognosis of cancer patients. Noticeably, we recently described that LRP-1 contributes to thyroid carcinoma cell invasion by subtly controlling the composition and turn-over of adhesive complex (9, 28).

Accumulating evidence now shows that LRP-1 may exert a pivotal function in the dynamic of cell-matrix interactions (28, 35, 36). Consistently, the receptor was demonstrated to influence several aspects of integrin functions critically engaged during aggressive cancer cells epithelial-to-mesenchymal transition, such as activation (8), clustering (37) and endocytosis that leads to either cell surface recycling or catabolism (10, 38–40). Tumor cells migration through extracellular matrix network is a complex multistep process relying on spatially and temporally regulated assembly of adhesion sites at the leading edge, cell contraction, and focal adhesion turn-over at the rear. Although LRP-1 now emerges as a putative regulator of each process (4, 35), the intracellular events it may solicit to control both adhesion complex formation and disassembly remain partially unraveled and controversial. The intracellular cysteine proteases calpains are well known regulators of cell migration for controlling focal adhesion stability through limited proteolysis of cytoskeletal components (41). The two best characterized members of the family, calpain-1 and calpain-2, are ubiquitously expressed and can be regulated by their endogenous inhibitor calpastatin or by various post-translational modifications, including autoproteolysis, calcium or phosphoinositide binding (41–43). In this study we pursued our investigation on LRP-1-dependent intracellular signals sustaining cell-matrix interactions dynamics by testing its contribution to calpain activity control in the context of thyroid carcinoma.

## Materials and methods

### Antibodies and chemicals

Anti-LRP-1 α-chain (8G1), anti-LRP-1 β-chain (5A6), anti-human IgGs used as negative control for immunoprecipitation experiments (HP6030), specific inhibitors for calpain (Calpeptin) and PKA (KT5720), and fluorigenic Calpain Substrate II were obtained from Calbiochem (distributed by VWR International, Strasbourg, France). Anti-calpain-2 (H240) used for immunoprecipitation experiments, anti-β1 (M-106), anti-calpastatin (C-20) and anti-β-actin (sc-1616) antibodies were purchased from Santa-Cruz Biotechnology (distributed by Tebu-Bio, Le Perray en Yvelines, France). Anti-phosphothreonine (9381) and HRP-conjugated anti-rabbit antibodies were from Cell Signaling (distributed by Ozyme, Saint Quentin Yvelines, France). Anti-calpain-2 (ab39165) and anti-talin (8D4) were from Abcam (Cambridge, UK). Anti-talin antibody used for immunocytochemistry (MAB1676) and anti-calpain-1 (MAB3104) were from Chemicon (distributed by Millipore, Molsheim, France). HRP-conjugated anti-mouse antibody (NA931V) was from Amersham Biosciences (Buckinghamshire, UK). Anti-pan β1 integrin (clone Mab13) and anti-active β1 integrin (9EG7) antibodies were from BD Transduction Laboratories (Mississauga, Canada). 7-amino-4-chloromethylcoumarin, t-BOC-L-leucyl-L-methionine amide (CMAC, t-BOC-Leu-Met), anti-mouse AlexaFluor 488 (A11001), AlexaFluor 568-phalloidin (A12380) and Prolong Gold antifade reagent with DAPI (P36935) were from Molecular Probes (Cergy Pontoise, France). Anti-phosphoserine (PSR-45), HRP-conjugated anti-goat secondary antibodies, leupeptin and other chemicals were purchased from Sigma-Aldrich (Steinheim, Germany).

### Cell culture and transfection

The FTC133 human follicular thyroid carcinoma cell line was grown in Dulbecco’s Modified Eagle Medium-Ham’s F12 (Invitrogen, Cergy Pontoise, France) with 10% FBS, as previously described (44). SiRNA mediated silencing of LRP-1 was described elsewhere (9). Specific LRP-1 targeting sequences were designed by Dharmacon (distributed by Perbio Science, Brebiere, France) as follows: GACUUGCAGCCCCAAGCAGUU (sense), CUGCUUGGGGUGCAAGUCUU (antisense). Control transfection experiments were achieved by using SiGENOME Non-targeting siRNA #1 (D00121001-20) from Dharmacon. For transient transfection assays, siRNA were transfected for four hours by using Lipofectamine 2000 (Invitrogen) according to manufacturer instructions.

### Reverse transcription and quantitative real-time PCR

Total RNAs isolated using the RNeasy Mini kit (Qiagen, Courtaboeuf, France) were reverse transcribed to cDNA with the ABsolute Blue verso 2-step kit and subjected to quantitative real-time PCR by using the Absolute SYBR Green mix (Thermo Fisher Scientific, Epsom, Surrey, UK) and a Chromo4 Real-Time PCR Detector system from Bio-Rad. Primers for LRP-1 were previously described (9). Primers for calpain 1, calpain 2 and β-actin were designed by Eurogentec (Seraing, Belgium) as follows: calpain 1: forward, CCTTGAGGATGATCTGGTAGA; reverse, AGCTAGTGTTCGTGCACTCTG; calpain 2: forward: CTGGAGATCTGTAACCTGACC; reverse: GGTACTGAGGGTTCATCCAGA; β-actin: forward: GTGTGACGTGGACATCCGC; reverse: CTGCATCCTGTCGGCAATG. The relative levels of expression were quantified by using Opticon Monitor software (Bio-Rad). For each specific gene, the amount of target RNA (2^-ΔΔCt^) was normalized to the internal actin reference (ΔCt) and related to the amount of target RNA in control sample, set to 1. All experiments were performed at least in triplicate with internal duplicate for each sample.

### Western blot analysis

Whole-cell extracts were prepared by scrapping cells in ice-cold lysis buffer (25 mM Tris-HCl, pH 7.7, 150 mM NaCl, 0.5% glycerol, 1% Triton X-114, 5 mM EDTA, 0.5 mM EGTA, 1 mM phenylmethylsulfonyl fluoride, 1 mM orthovanadate supplemented with proteinase inhibitor cocktail). After 30 min incubation on ice, extracts were centrifugated (10,000 X *g* for 10 min at 4°C) and pellets were discarded. The protein concentration in supernatants was quantified by the Bradford method (BioRad Laboratories). Proteins were separated by electrophoresis in a 10% sodium dodecyl sulfate-polyacrylamide gel, transferred onto a nitrocellulose membrane (Whatman GmbH, Dassel, Germany), and incubated overnight at 4°C with primary antibodies. Immunoreactive bands were revealed using an ECL plus chemiluminescence kit from Amersham Biosciences by using a ChemiDoc-XRS imaging station from Bio-Rad. Immunoblots presented are representative of at least three separate experiments. The specific signal of β-actin was used to ensure equal loading.

### Biotinylation of cell surface proteins and detection of cell surface integrins

Separation of cell surface proteins was conducted by immunoprecipitation of biotinylated proteins. Briefly, after culture on gelatin-coated surfaces, cells were rinsed with ice cold PBS and incubated for 30 min at 4°C under slow agitation with 0,5 mg/mL EZ-Link Sulfo-NHS-LC-Biotin (Pierce). All further steps until cell lysis were made at 4°C to avoid cell surface protein endocytosis. After 3 PBS washes, reaction was quenched by 100 mM Glycine for 30 min under gentle agitation and cells were scrapped in lysis buffer (10 mM Tris-HCl, pH 7.4, 150 mM NaCl, 0,1% Triton X-100, 5 mM EDTA, 1 mM phenylmethylsulfonyl fluoride, 1mM orthovanadate supplemented with proteinase inhibitor cocktail). 500 µg of clarified cell extracts were then incubated for 1 hour with 40 µL of avidin beads (Pierce) under agitation. After three washes in lysis buffer, immunoprecipitated proteins were reduced and analyzed by immunoblotting. Supernatants were used to control selective fractionation of cell surface proteins. When indicated, cell surface specific integrin-containing complexes were subjected to a second immunoprecipitation step after competing biotin-avidin complexes overnight with 10 mM D-Biotin (Pierce). Procedure for immunoprecipitation is described in the following section.

### Immunoprecipitation

The procedure was adapted from one previously described (9). Briefly, for the immunoprecipitation of cell surface integrin containing complexes, Biotinylated protein fraction was subjected to a second immunoprecipitation step in the same lysis buffer, as described above. Cell extracts for immunoprecipitation of calpain were prepared in a distinct lysis buffer (10 mM Tris-HCl, pH 7.4, 0.75% Brij, 75 mM NaCl, 5 mM EDTA, 1 mM phenylmethylsulfonyl fluoride, 1 mM orthovanadate supplemented with proteinase inhibitor cocktail). After centrifugation (10,000 X *g*, 10 min at 4°C) and Bradford titration, 500 µg of total proteins were incubated for 4 hours at 4°C on an orbital agitator in presence of anti-calpain antibodies. Nonspecific IgGs were used for negative controls. Immunoprecipitation was performed with 40 µL protein G-Sepharose (Amersham Biosciences) for 2 hours at 4°C under agitation. The samples were washed three times in the corresponding lysis buffer and protein complexes bound to beads were solubilized under reducing conditions and analyzed by immunoblotting as described above.

### PKA activity assay

PKA activity was assessed by using ProFluor PKA assay from Promega with minor modifications to quantify specific PKA activity in raw cell extracts. Briefly, cells were detached and cultivated on gelatin-coated for 3 hours. Cells were then scrapped 48 hours post transfection on ice in PKA buffer (25 mM Tris-HCl, pH 7.4, 0,5 mM EDTA, 0,5 mM EGTA), sonicated, clarified (10,000 X *g* for 10 min at 4°C) and protein concentration was determined by Bradford method. Equal amounts of proteins were then assessed for PKA activity following standard procedure, based on phosphorylation-dependent quenching of a fluorescent PKA substrate. Results were obtained from three separate experiments with internal quadruplicate, and related to control sample, set to 1. Specificity of reaction was ensured by subtracting residual PKA activity in the presence of 10 µM of specific inhibitors H-89 and KT5720.

### Calpain activity assays

Cells were scrapped on ice in lysis buffer (50 mM Hepes, pH 7.4, 150 mM NaCl, 1% Triton X-100, 0,5 mM DTT, 10 mM β-mercapto-ethanol, 20 µM leupeptin, 1 mM PMSF, 1 mM Na_3_VO_4_) and clarified (10,000 X *g* for 10 min at 4°C). *In vitro* calpain activity was then quantified through two different procedures. First, calpain activity was measured with Innozyme Calpain 1/2 Activity Assay Kit (Calbiochem) following manufacturer instructions. A second procedure was adapted from Goette *et al.* (45). Briefly, 100 µg of proteins were diluted in activation buffer (20 mM Tris-HCl pH 7.4, 5 mM CaCl_2_) containing 2 µM calpain substrate II (Suc-Leu-Tyr-AMC). Background fluorescence was determined by negative control carried out for each sample in the presence of 10 mM EDTA, 10 mM EGTA and 50 µM of the specific calpain-1/2 inhibitor calpeptin. Reaction was developed in 96-wells plate at 37°C for 1 hour and fluorescence resulting from calpain-mediated release of AMC was measured at 380 nm (excitation)/430 nm (emission) wavelengths using a Perkin-Elmer LS50B spectrometer. Results were expressed as percentage of control after background subtraction. Each experiment was made at least four times with internal quadruplicate. Calpain activity was visualized in intact cells by using a previously described method (46). Cells were cultivated on gelatin-coated coverslips and incubated for 10 min in the presence of 50 µM BOC-LM-CMAC (*t* butoxycarbonyl-Leu-Met-chromomethylaminocoumarin), a cell permeant thiol-reactive substrate selective for calpains. Cells were then replaced in fresh medium for 1 hour, fixed on ice in 4% paraformaldehyde and mounted in aqueous antifading medium (Immunotech). Fluorescence resulting from Calpain-mediated CMAC release was observed under UV light with an optical microscope (BH2; Olympus). Specificity of the reaction was controlled in the presence of 50 µM calpeptin. Quantification of calpain activity was achieved by counting 300 cells from 3 distinct experiments. The number of positive cells exhibiting a clear fluorescence signal was normalized to a total cell number for each treatment and expressed as a percentage of control condition.

### Boyden chamber assay

Matrigel invasion assay was performed using modified Boyden chambers in 24-well dishes with filter inserts provided with 8-µm pores (Transwell, Costar, Brumath, France), as described elsewhere (44). Matrigel-coated filters were used for invasion assays and uncoated filters were used for migration assays. Invasiveness was determined by counting cells in eight random microscopic fields per well, each seeded in triplicate.

### Adhesion and trypsinization assays

Measurement of cancer cell adhesion to gelatin-coated surfaces and trypsinization assays were carried out according to a previously described method (9).

### Immunofluorescence microscopy

FTC133 cells were seeded onto gelatin-coated coverslips for 2 hours at 37°C, fixed with 4% paraformaldehyde for 5 min on ice and then permeabilized in ice-cold 0,1% Triton X-100 for 10 min. After three washes with PBS, cells were incubated for 30 min with PBS containing 2% bovine serum albumin to saturate nonspecific antigens. Coverslips were then incubated for 30 min with AlexaFluor 568-phalloidin or 60 min with anti-talin antibodies followed by three washes with PBS (Phosphate Buffered Saline). Talin staining was revealed after 30 min incubation with secondary antibodies conjugated to AlexaFluor 488. Coverslips were then mounted in Prolong Gold antifade medium with DAPI to obtain nuclei counterstaining. All acquisitions were made with an Olympus BH2-RFCA epifluorescence microscope (Olympus, Japan), equipped with a 100W mercury lamp (OSRAM, GmbH), using a SPlan achromat x 40 objective (Olympus) and fluorescein, rhodamin, DAPI filters for talin, actin and nuclei staining, respectively. Antibodies used for immunostainings of integrins were incubated overnight and revealed after 1 hour incubation with Alexafluor 568-coupled secondary antibodies. Images were acquired with Zen software on a Zeiss LSM 880 Airyscan confocal microscope (Oberkochen, Germany) using a x63 Plan Apochromat objective (oil immersion, 1.40 NA). Representative images from three separate sets of cultures were treated and merged with ImageJ software. The percentage of cells positive for focal adhesions was determined as previously reported (9), by examining 300 cells from three different experiments for each condition.

### Densitometric analysis and statistical evaluation

Bands from immunoblots or agarose gels were quantified by using Quantity One image-analyzer software (Bio-Rad Laboratories). All culture assays were normalized on the basis of cell viability by using the CellTiter-Glo assay from Promega. Each experiment was performed at least in triplicate and data were expressed as mean ± SEM. Comparisons between two or four samples were performed using Student’s t-test or one way analysis of variance (47) with *post hoc* Tukey’s test (Prism software, GraphPad Inc, San Diego, CA).

## Results

### LRP-1 expression contributes to repress calpain activity in invasive thyroid carcinoma cells

**Figure 1:**
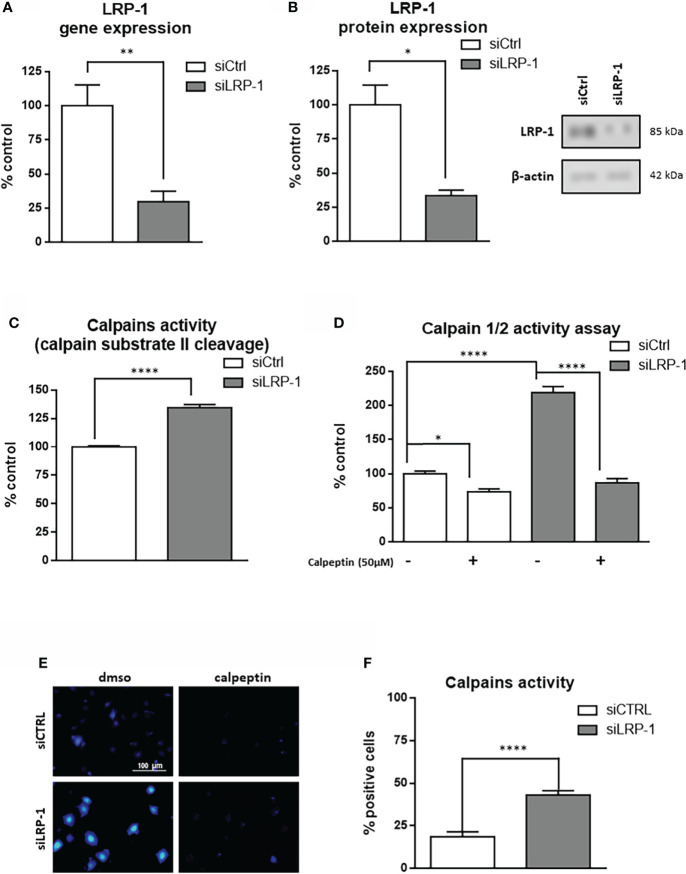
LRP-1 controls calpain activity in carcinoma cells. FTC133 cells were transfected with siRNA against LRP-1 expression (siLRP-1) or non-targeting control sequences (siCtrl) and total RNA were extracted after 48 hours. The transcriptional level of LRP-1 expression was assessed by RT-qPCR, using β-actin as a normalization control **(A)**. The LRP-1 protein expression was assessed by western blot, using β-actin as a normalization control. A representative picture of immunoblots is presented **(B)**. Whole extracts FTC133 cells transfected with siRNA sequences were used to test their ability to degrade the calpain substrate II, measured by spectrofluorimetry. Results are expressed as percentage of control after background fluorescence subtraction **(C)**. Same extracts were used to measure calpain activity by using Innozyme Calpain 1/2 Activity Assay Kit in the presence or absence of 50µM of calpeptine to ensure the specificity of the reaction **(D)**. Intracellular activity of calpains was visualized by fluorescence microscopy in intact cells treated with cell permeant calpain substrate BOC-LM-CMAC **(E)**. The percent of cells harboring strong intracellular accumulation of fluorescent CMAC, above fluorescent developed in presence of 50µM of calpeptin, was quantified **(F)**. Each value is mean ± sem for at least four independent experiments, each performed in triplicate. *, *P* < 0,05; **, *P* < 0,01; ****, *P* < 0,0001.

We previously reported that LRP-1 is involved in the regulation of cell-matrix interactions turnover by exerting an intimate control on the focal adhesion composition and dynamics (9, 28, 40). We demonstrated that LRP-1 functions as a molecular relay and provides an intracellular docking platform to regulate the incorporation and activation of MAPK into focal complexes. This work established a direct link between intracellular signaling pathways modulated by LRP-1 and dynamics of matrix attachment of migrating tumor cells. However, a complete overview of the intracellular connections of the receptor in that context is still lacking. We further investigated the still incomplete knowledge of intracellular functions of the receptor involved in the control of adhesion complexes stability. To achieve this goal, we used a validated method of invalidation of LRP-1 in FTC-133 thyroid carcinoma cells by siRNA (36) leading to a 70% decrease of its gene expression as evaluated by RT-qPCR **(** **Figure 1A** **)**, and to a 66% decrease of its protein expression measured by western blot **(** **Figure 1B** **)**. Calpains constitute a family of intracellular calcium-dependent cysteine-proteases which exert crucial actions on focal adhesion dynamics, and are able to cleave cytoskeleton components to mediate turnover and disassembly of focal complexes as well as the activation and clustering of extracellular matrix receptors (41, 48, 49). We tested the consequences of LRP-1 invalidation on calpain activity in FTC133 cells during the phase of attachment by performing a biochemical assay based on calpain substrate II cleavage **(** **Figure 1C** **)**. We evidenced an increase of 34,5% of the capacity of LRP-1-silenced cells to degrade calpain substrate II *in vitro*, as compared to control tumor cells. A distinct fluorigenic assay was conducted to confirm this result using the quenching of DABCYL substrate. By using this complementary approach, we observed a 2.2 fold increase of calpain activity in LRP-1-depleted cells, as compared to control **(** **Figure 1D** **)**. Of note, we were able to efficiently inhibit calpain activity by adding 50µM of their specific inhibitor calpeptin. A 26% inhibition was detected in control cells whereas the amplitude of this inhibition in LRP-1-silenced cells was 60%, reinforcing the idea that the baseline activity of this class of proteases is significantly higher in the absence of the receptor. Finally, we incubated thyroid carcinoma cells with the cell permeant substrate of calpains BOC-LM-CMAC in order to visualize their activities in living cells **(** **Figure 1E** **)** (46). We evaluated the proportion of cells exhibiting high calpain-dependent release of fluorescent CMAC probe **(** **Figure 1F** **)**. In LRP-1 expressing control cells, 18,6% of the total cells were fluorescent when 43% of LRP-1 depleted cells had developed the calpain-dependent reaction. In conclusion, we showed by three independent biochemical assessments that LRP-1 silencing leads to a significant induction of intracellular calpain activity in thyroid carcinoma cells.

### LRP-1-dependent attenuation of calpain activity contributes to migratory capacities of carcinoma cells by regulating cell-matrix attachment processes

**Figure 2:**
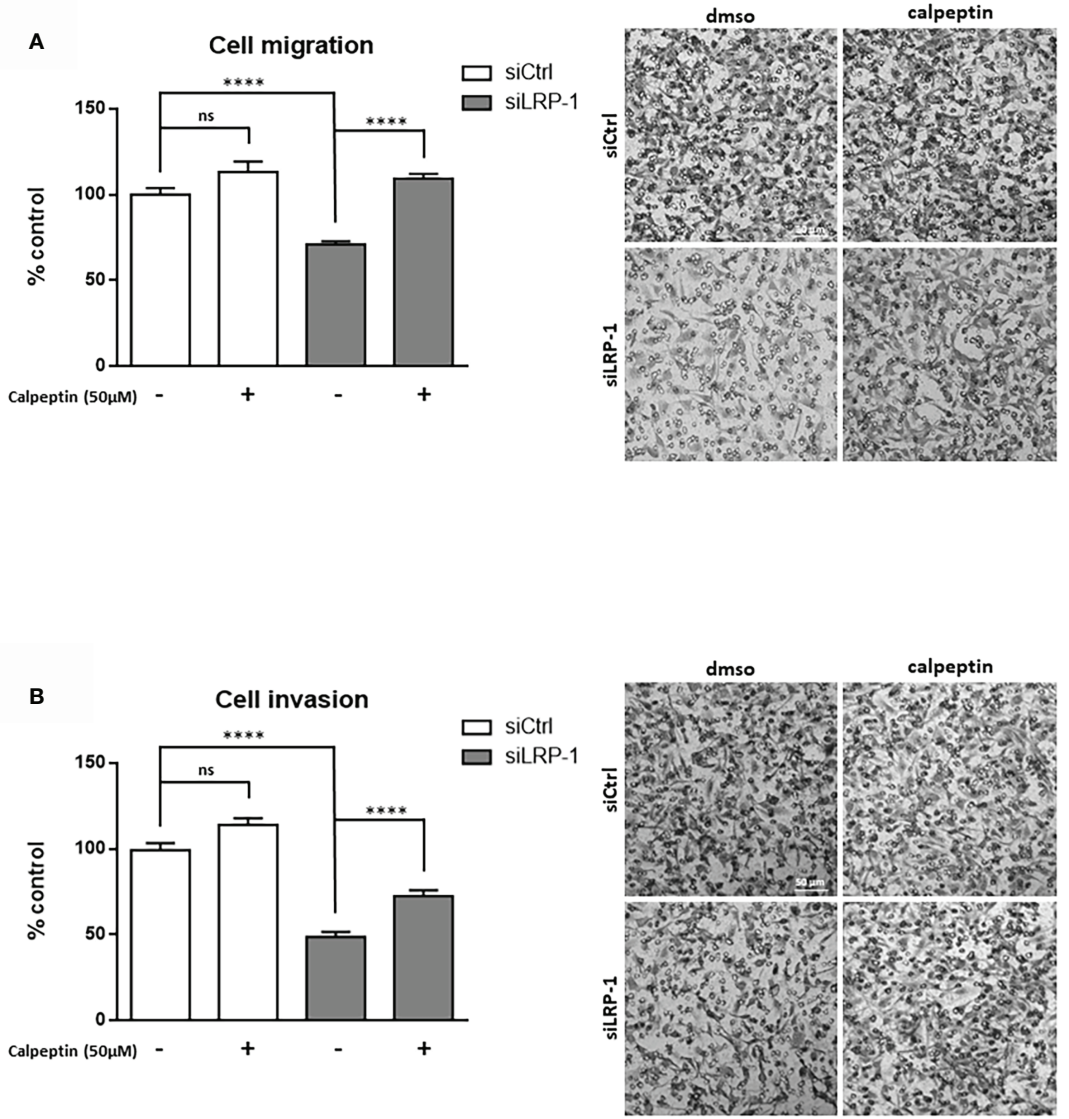
LRP-1-mediated control of calpain activity contributes to carcinoma cells migratory and invasive properties. Cell migration and invasion assays were carried out with FTC133 cells transfected with siRNA sequences, treated with 50 µM calpeptin or vehicle. Three-dimensional cell migration was assessed by using uncoated Transwell filters **(A)**. Tumor cell invasion was measured on Matrigel-coated Transwell membranes **(B)**. Representative images are shown. Migration and invasion were determined by counting cells in eight random microscopic fields per well. Results are expressed as mean ± sem after normalization by comparison with siCtrl transfected cells set to 100%, from at least four distinct experiments each performed in triplicate. ns, not significant; ****, *P* < 0,0001.

**Figure 3:**
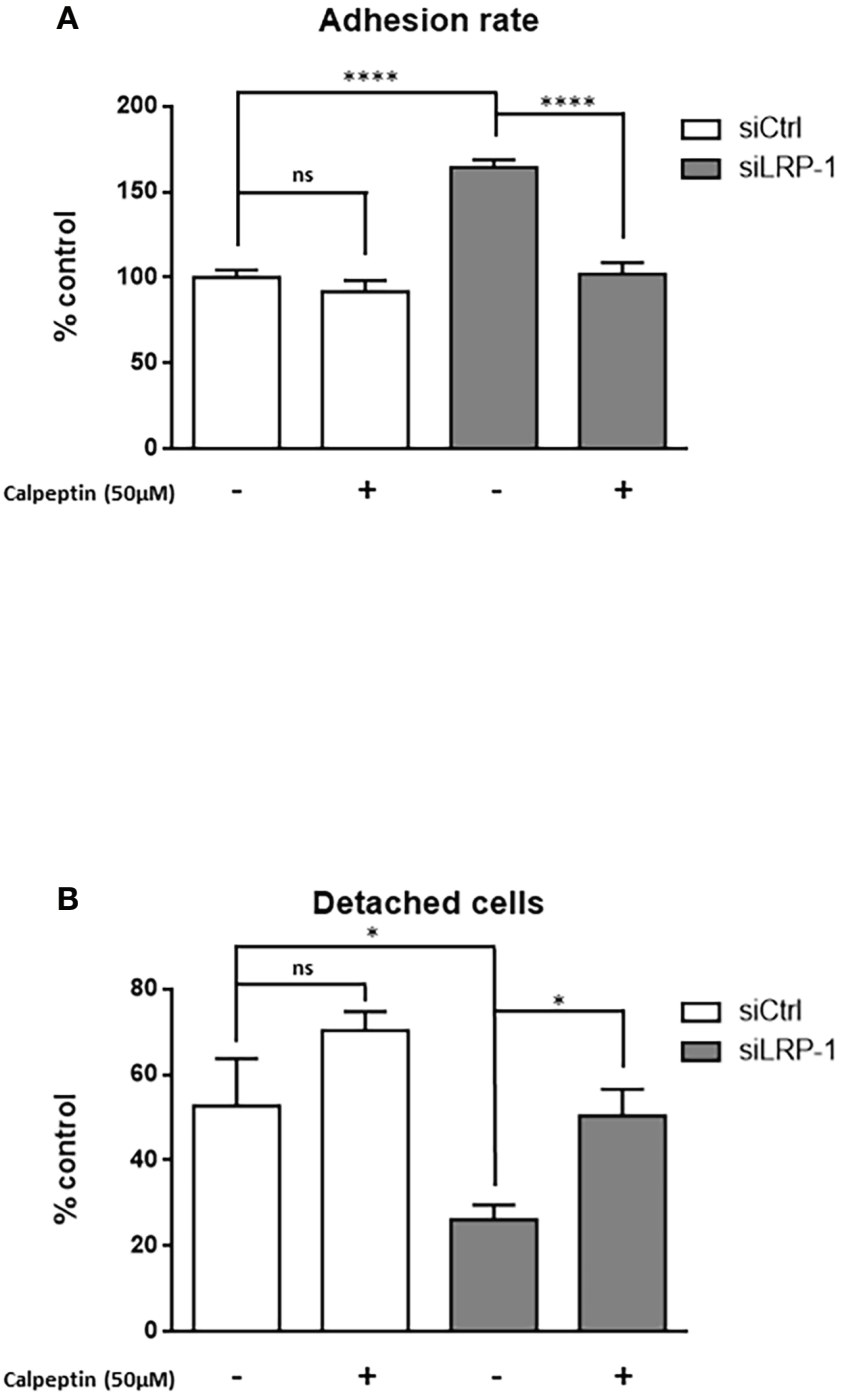
Calpain activity modulates carcinoma cells adhesion strength. FTC133 cells transfected with siCtrl or siLRP-1 RNA were seeded onto gelatin-coated plates in presence or absence of 50µM calpeptin, and the non-adherent cells were discarded after 60 minutes **(A)**. Transfected tumor cells were grown in gelatin-coated dishes for 24 hours and subjected to trypsinization assay by incubating cells with 0,025% (wt/vol) trypsin for 10 minutes **(B)**. For each cell condition, results are expressed as mean percentages of attached or detached cells ± sem, relative to control set to 1. ns, not significant; *, *P* < 0,05; ****, *P* < 0,0001.

We reported that LRP-1 functionalities in invasive cancer cells are not restricted to the endocytic control of extracellular matrix degrading enzymes (9). We showed that this endocytic receptor can directly feed pro-motile signals by coordinating the stability and distribution of cell surface matrix receptors through its control of downstream intracellular signaling events (28, 36, 39). In order to measure the consequences of the LRP-1-mediated control of calpain activity, we performed Boyden chamber assays with FTC133 cells LRP-1-silenced or not, in the presence or absence of the specific calpains inhibitor calpeptin **(** **Figure 2A** **)**. As expected, we observed that LRP-1 silencing reduced carcinoma cell migration by 29% as compared to migrated control cells. Interestingly, addition of 50µM calpeptin completely restored the migration of LRP-1-silenced cells to the level of control cells. The same treatment did not exert any significant effect on LRP-1 expressing cells, suggesting that calpain activity is low. We conducted complementary modified Boyden chamber assays to analyze the invasive behavior of thyroid carcinoma cells using Matrigel as matrix barrier **(** **Figure 2B** **)**. In that context, the consequences of LRP-1 depletion were more drastic since the proportion of invading cells was decreased by 51% in LRP-1 deficient-cells. Inhibition of calpain activity in LRP-1-depleted cells restored invasive potential that recovered 71% of the level observed in control cells. In contrast, the calpain inhibitor failed to modulate the invasive ability of LRP-1 expressing cells. We therefore demonstrated that LRP-1 maintains a low level of calpain activity that contributes to the migratory and invasive capacities of carcinoma cells. We then conducted adhesion assays to test the likely involvement of LRP-1-dependent calpain regulation in the control of carcinoma cell attachment to matrix compounds **(** **Figure 3A** **)** (28). We evidenced an increase of the adhesion rate of cells lacking LRP-1, as 64% more cells spread compared to control cells. Inhibition of calpain activity reduced the adhesion rate of LRP-1-silenced cells to the level of control situation. By contrast, the adhesion of LRP-1 expressing cells adhesion was not affected by calpeptin treatment. In a complementary experimental set-up, we performed detachment assays of fully spread carcinoma cells in response to the enzymatic action of trypsin, using the proportion of cells remained adherent as a readout for cell-matrix interactions strength (36). The proportion of detached cells after LRP-1 knock-down was decreased by 51% as compared to control cells **(** **Figure 3B** **)**. Adding calpeptin during the previous phase of attachment reduced the resistance of LRP-1-silenced cells to trypsin to recover the detachment rate detected in control cells. Although a similar trend could be observed in LRP-1 expressing control tumor cells, it was not significant. Together, those data illustrated that the pro-invasive action of LRP-1 in thyroid carcinoma cells relies in part on the maintenance of a low level of calpain activity leading to a moderate cell-matrix interactions strength.

### LRP-1 limits transcriptional expression of calpain-2 and inhibits calpain activity through a PKA-dependent mechanism

**Figure 4:**
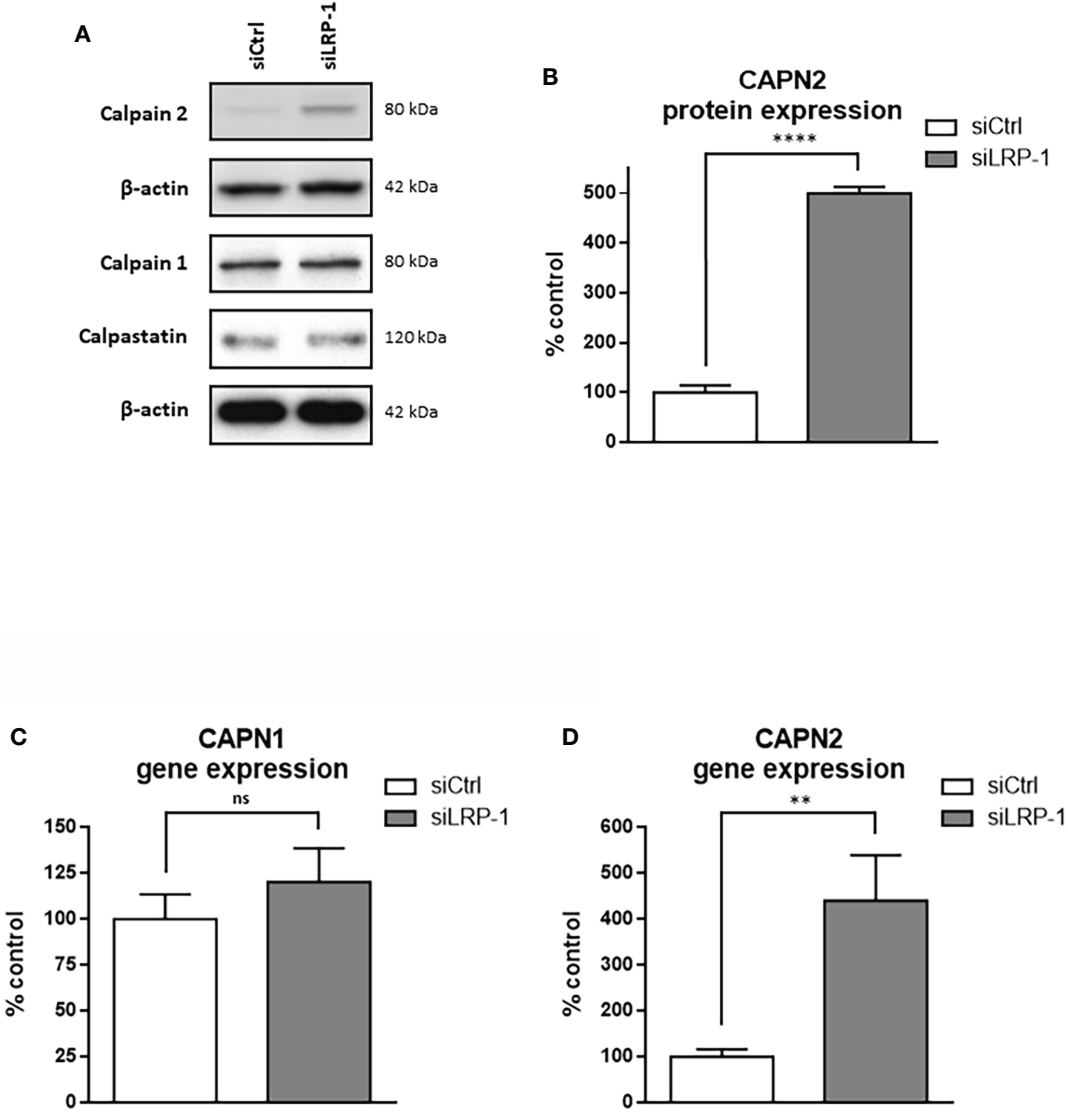
LRP-1 maintains low level of CAPN2 in carcinoma cells. Whole extracts from FTC133 cells transfected with siRNA sequences were analyzed by SDS-PAGE and western blot using specific antibodies against calpain-2, calpain-1, calpastatin and β-actin that was used as loading control. Pictures are representative of 3 independent experiments **(A)**. Densitometric analysis of calpain-2 specific bands normalized to actin signal and expressed as percentage of siCtrl transfected cells **(B)**. The transcriptional levels of calpain-1 **(C)** and calpain-2 **(D)** expressions were assessed by RT-qPCR, using β-actin as a normalization control. Results shown are expressed as mean ± sem percentage of the expression measured in control cells from 6 independent experiments. ns, not significant; **, *P* < 0,01; ****, *P* < 0,0001.

**Figure 5:**
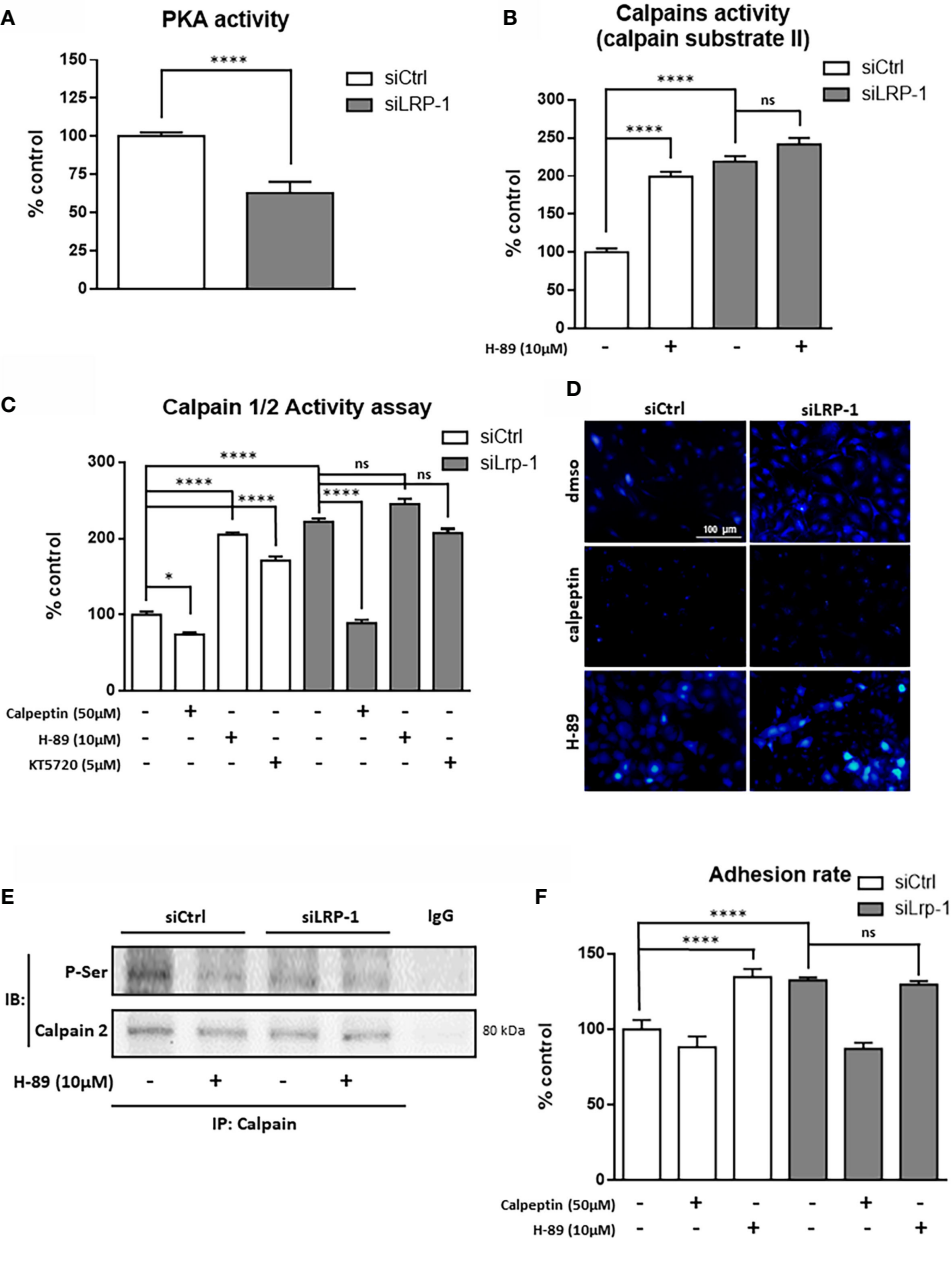
LRP-1-dependent PKA signaling limits CAPN2 activity. PKA activity was measured in whole extracts of FTC133 cells transfected with siRNA sequences and cultivated 3 hours on gelatin-coated dishes by using the fluorigenic ProFluor PKA enzymatic assay (Promega), adapted to measure specifically PKA activity in raw cellular extracts **(A)**. Extracts from FTC133 cells, treated with 10µM H-89 (PKA inhibitor) or vehicle, were used to measure the calpain specific degradation of the fluorigenic calpain substrate II **(B)**. Same extracts were used to test the impact of PKA on calpain activity measured by the Calpain ½ Activity Assay Kit in presence or absence of 50µM of calpeptin, 10µM of H-89 or 5µM of KT5720 **(C)**. Calpains-specific release of CMAC from calpains substrate BOC-LM-CMAC was analyzed in intact cells after treatment with calpeptin of H-89 **(D)**. Whole-cell extracts were isolated from FTC133 cells treated with 10µM H-89. Co-immunoprecipitation of calpain-2 were performed and phosphorylation status of the protease was analyzed by SDS-PAGE and immunoblotting using an antibody reacting with phosphorylated serine residue **(E)**. FTC133 cells transfected with siCtrl or siLRP-1 RNA were seeded onto gelatin coated plates in presence or absence of calpeptin or H-89 and the non-adherent cells were washed out after 60 minutes **(F)**. Results are representative of at least 3 independent experiments and are expressed as mean percentage from a minimum of 4 internal replicates standardized to control value. ns, not significant; *P < 0,05, ****, *P* < 0,0001.

Having identified calpains as new intracellular targets regulated by LRP-1 in carcinoma cells, we next set up an experimental design to decipher the mechanistic nature of the process controlling their activities. First we evaluated the differential expression of members of the ubiquitous calpain family by western blot **(** **Figures 4A, B** **)**. Although we did not observe any difference of expression neither of calpain-1 (µ-calpain) nor of calpastatin, the endogenous inhibitor of activated calpains, we evidenced a significant 5-fold increase of calpain-2 (m-calpain) protein expression in LRP-1-silenced FTC133 cells. We then analyzed the level of the transcriptional expression of the two main proteases, namely calpain 1 and calpain-2, by RT-qPCR **(** **Figures 4C, D** **)**. Consistently, calpain-1 transcriptional level was not affected by LRP-1 modulation, whereas calpain-2 gene expression was 4,4-fold increased under LRP-1 silencing. Since the expression level of calpastatin was not altered according to the expression of LRP-1, we then focused our attention on the regulation of the calpain activity by phosphorylation. In our screen for potential LRP-1 downstream signaling events, we measured PKA activity in cellular extracts from both LRP-1-silenced and control FTC133 cells. We evidenced that LRP-1 inhibition led to a 1.6-fold decrease of PKA activity **(** **Figure 5A** **)**. Interestingly, PKA-dependent phosphorylation of calpain-2 had already been reported to inhibit the proteolytic activity of ubiquitous calpains (46). Accordingly, the treatment of FTC133 cells with H-89, a specific inhibitor of PKA, induced a 2-fold increase of calpain activity as measured by the cleavage of calpain substrate II **(** **Figure 5B** **)**. H-89 treatment did not significantly affect this activity when LRP-1 was silenced. To support these data, we then used two distinct inhibitors of PKA activities, H89 and KT5720, and evaluated the impact on calpain activity using the fluorogenic calpain activity assay described in **Figure 1D** **(** **Figure 5C** **)**. We evidenced that H-89 or KT5720 treatment respectively resulted in about a 2-fold and 1.7-fold increase of basal calpain activity in LRP-1 expressing carcinoma cells. Inhibition of PKA by H-89 was also efficient to increase the proportion of FTC133 cells positive for calpain activity **(** **Figure 5D** **)**. Importantly, this effect was specific for controlling cells in which PKA activity was mainly detected. We then conducted pull-down experiments to study the phosphorylation status of calpain-2 **(** **Figure 5E** **)**. We were able to immuno-precipitate the protein and we evidenced by western blot that its level of phosphorylation on serine residues was higher in control than in LRP-1 depleted cells. This signal was decreased in the presence of H-89, indicating that PKA-dependent mechanism was in part responsible for serine phosphorylation of calpain-2 in FTC133 cells. Adhesion assays were also conducted to demonstrate that PKA activity was functionally associated to the control of carcinoma cell attachment and spreading by LRP-1-mediated regulation of calpains **(** **Figure 5F** **)**. Indeed, we specifically showed that inhibition of PKA by H-89 treatment in LRP-1 expressing cells enhanced their adhesion rate to gelatin to a similar level to that observed after LRP-1 inhibition. Our data indicated that PKA helped to limit calpain activity in a LRP-1-dependent manner in the frame of cell-matrix interactions established by thyroid carcinoma cells.

### Regulation of calpain activity by LRP-1 plays a part in adhesion complexes lability and limits cell surface β1-integrin distribution

**Figure 6:**
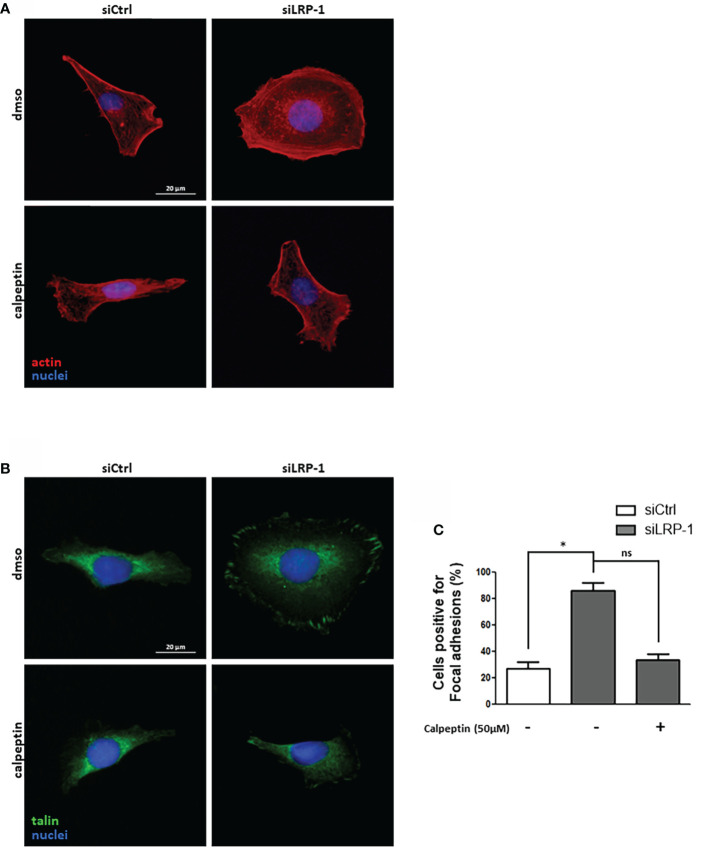
LRP-1-mediated control of calpain activity fine-tunes spreading and focal adhesion maturation in carcinoma cells. FTC133 cells transfected with siRNA sequences were cultivated two hours on gelatin-coated coverslips in the presence or absence of 50µM of calpeptin, fixed, and labelled with phalloidin to analyze their morphology and fibrillar actin network (red) by fluorescence microscopy. Nuclei were counterstained with DAPI (blue) **(A)**. Alternatively, cells were immunostained with an antibody against talin (green) to analyze the formation of focal adhesion **(B)**. The percentage of cells positive for focal adhesions was quantified following the inhibition of calpain activity by calpeptin or control treatment **(C)**. For each condition, at least one hundred of cells from three separate experiments were evaluated. ns, not significant; *, *P* < 0,05.

**Figure 7:**
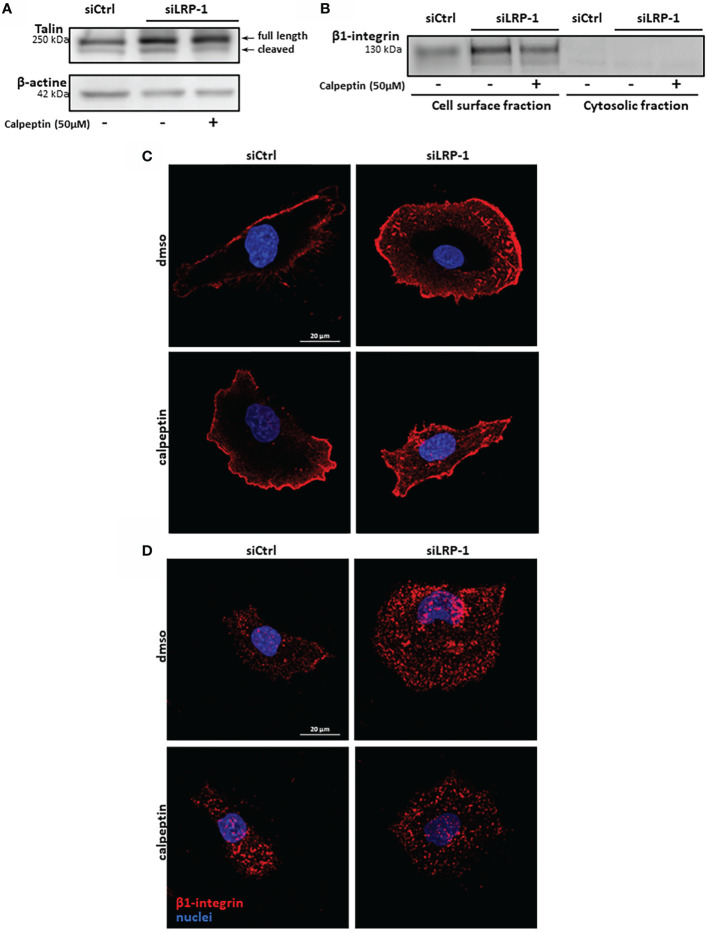
β1 integrin membrane expression is controlled by LRP-1-dependent calpains regulation. Whole extracts from FTC133 cells transfected with siRNA sequences, seeded onto gelatin and cultivated in the presence or absence of calpeptin for 3 hours were analyzed by SDS-PAGE and western blot using specific 8D4 antibodies against talin, allowing the detection of native full length and calpain-released cleaved product of talin. β-actin that was used as loading control. Picture is representative of 3 independent experiments **(A)**. FTC-133 cells were cultivated in the same conditions and biotinylated at 4°C to label cell-surface proteins. Membrane fraction was purified by precipitation of surface proteins by using avidin-sepharose beads. Surface expression of β1-integrins was analyzed by SDS-PAGE and immunoblotting. Corresponding cytosolic fractions that were not immobilized by avidin beads were loaded as a control of cell membrane purity **(B)**. Tumor cells transfected with siCtrl or siLRP-1 RNA were seeded onto gelatin coated coverslips for 2 hours in the presence of calpeptin and immune-stained with antibodies directed against either total β1-integrins pool **(C)** or active forms of the receptor **(D)**. Confocal microscope captures in Z series were acquired and projected by using maximum intensity, stacking the 10 optical slices attached to the coating. Pictures are representative of 3 separate experiments.

Considering the deep consequences of LRP-1-dependent regulation of calpain activity on the invasive potential and adhesion/deadhesion processes of tumor cells, we then analyzed the distribution and the morphology of cytoskeleton constituents. The fibrillar actin network of thyroid carcinoma cells after two hours of attachment was characteristic of spread and polarized cells such as mesenchymal cells **(** **Figure 6A** **)**. Most of the fibrillar structures positively stained by phalloidin were concentrated at the cell periphery and protrusion sites. This specific pattern was not modified by calpains inhibition in control cells. In accordance with previous reports (9, 28), LRP-1 silencing induced drastic changes of carcinoma cells morphology and actin fibers distribution. Cells were overspread as compared to control cells and exhibited prominent radial and transverse fibers, widely distributed, both at tumor cell center and periphery. Under LRP-1 inhibition the calpain inhibitor induced a clear transition of cells toward the mesenchymal and elongated aspect observed in the control condition. We performed talin immuno-fluorescent staining to evaluate the consequences of calpains inhibition. In accordance with the morphological transitions described above, calpeptin did not affect the proportion of talin-positive structures in control cells, whereas LRP-1 inhibition reinforced the proportion of cells exhibiting obvious and stabilized talin-positive structures **(** **Figure 6B** **)**. The occurrence of these complexes was specifically decreased in LRP-1-depleted cells under calpeptin treatment **(** **Figure 6C** **)**. Our data illustrated that during the attachment of FTC133 cells to gelatin, calpains inhibition by LRP-1 sustained the polarization of thyroid carcinoma cells and constituted a limiting mechanism in focal adhesion maturation. We then measured the protein expression of talin by western blot using the 8D4 antibody, allowing the detection of the full length protein and its calpain-dependent cleavage fragment **(** **Figure 7A** **)**. In accordance with the altered distribution of talin observed by immunocytochemistry **(** **Figure 6B** **)**, we detected an accumulation of both native and cleaved forms of talin in LRP-1-silenced tumor cells. Interestingly, calpeptin treatment appeared to limit the accumulation of the cleaved form of talin. In order to assess the level of adhesion receptors expressed at cell surface, we performed biotin labelling of tumor cells spread on gelatin and treated with calpain inhibitor or vehicle. Precipitated protein membrane fractions were analyzed by immunoblotting **(** **Figure 7B** **)**. Interestingly, we observed an increase of β1 integrin subunit at cell surface upon LRP-1 repression. This accumulation seemed partially decreased under calpeptin treatment. We then conducted immunostainings to qualify the distribution of β1 integrins in non-permeabilized carcinoma cells cultivated in the same conditions **(** **Figure 7C** **)**. β1 integrins were detected in FTC133 cells and were mostly present at the periphery and at the front of spread and elongated cells. The presence of β1 integrins was accentuated in LRP-1 repressed cells and highly accumulated along the whole cell periphery. In the presence of calpeptin the polarized morphology of control cells did not show any clear critical transition, in opposition to LRP-1-silenced tumor cells which were more frequently polarized and spindle shaped and exhibited a discontinuous β1 integrin-positive margin that appeared more concentrated on distal sites of cell projections. We then conducted immunostainings by using an antibody specific for active forms of β1 integrins **(** **Figure 7D** **)**. The localization of these forms of the receptors in non-permeabilized cells was more punctuated with some positive clusters at the leading edge of elongated control cells. These clusters were evenly observed across the ECM-attached area from overspread LRP-1-silenced cells. After inhibition of LRP-1 expression, both the number and size of theses clusters were diminished upon calpeptin treatment when LRP-1 was down-regulated. Altogether, these data showed that LRP-1-mediated control of calpain activity determined cell surface distribution of β1 integrins in thyroid carcinoma cells.

## Discussion

**Figure 8:**
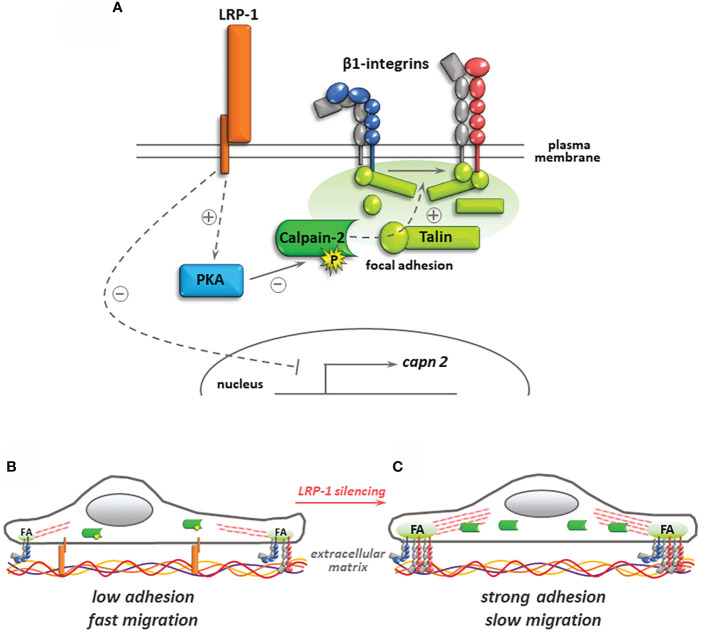
Regulation of calpain activity by LRP-1 and its consequences for thyroid carcinoma cell adhesion. In the presence of LRP-1, low transcriptional expression and PKA-mediated phosphorylation of calpain-2 controls talin-rich focal adhesion complexes dynamics and surface expression of inactive (blue) and active (red) forms of β1 integrins **(A)**. The resulting intermediate adhesive state contribute to the invasive potential of polarized tumor cells **(B)**. Under LRP-1 silencing, gene induction of calpain-2 and decrease of its PKA-dependent phosphorylation result in strong calpain activity. Subsequently, tumor cells exhibit an overspread phenotype explained by surface accumulation of β1 integrin and focal adhesion (FA) stabilization leading to impaired invasive capacities **(C)**.

In the present study, we identified for the first time the regulation of calpain activity as a new mechanism by which LRP-1 controls the spreading and migratory capacities of thyroid carcinoma cells. Indeed, we have shown that LRP-1 restrains calpain activity, both through a transcriptional mechanism targeting capn2 gene expression, but also *via* PKA activation and Ser-phosphorylation on calpain-2. Such a regulation contributes to the achievement of an intermediate and optimal attachment state and promigratory phenotype **(** **Figure 8** **)**.

Our comprehension of LRP-1 contribution to cell-matrix interactions dynamics has significantly evolved in the past decade. Several studies revealed that this endocytic receptor controls cell surface stability and internalization of multiple cell surface complexes engaged during movements of migrating cells within their microenvironment, including matricellular proteins, integrins and proteoglycans (4, 28, 35, 36). This revised model of LRP-1 functionality could have been drawn earlier from studies showing that even proteolytic ligands of the receptor such as tissue plasminogen activator (tPA), bound to its inhibitor PAI-1, could trigger a full adhesion/deadhesion cascade of events when specifically complexed to LRP-1, fibronectin and LFA-1 (αLβ2) integrin (10). Proteomic and coimmunoprecipitation approaches then illustrated that LRP-1 acts as a *bona fide* adhesion receptor by itself, notably by recruiting to the intracellular tail of its β-chain focal adhesion and cytoskeleton components such as talin, paxillin or α-actinin (21, 28). These data support the idea that LRP-1 integrates extracellular and intracellular events and acts as a mechanosensing hub driving the fate, lifespan and turnover of nascent adhesion in a given cellular context. This concept was especially well documented for integrin-based adhesion to the extracellular matrix. After ligand binding, LRP-1 was involved in the reinforcement of cell surface expression of β1 integrins, and in their activation and signaling through integrin-linked kinase (8). LRP-1 was also identified as controlling tissue transglutaminase endocytosis, complexed with β1 integrin and fibronectin (38). We recently evidenced that the association of β1 integrin with domain II and IV of LRP-1 extracellular α-chain in thyroid carcinoma cells exerted a crucial function in routing it in a Rab11 endocytic recycling traffic (39). A parallel study showed that LRP-1 was engaged in the association with the intracellular domain of β1 integrin through kindlin-2 (40), a family of adhesion scaffolds that also constitute cleavage substrates for calpains (50). Most of the published litterature illustrated the de-adhesive consequences of the complexe interactions of LRP-1 with integrins (10, 38, 39). The control of cell surface expression and activation of β1 integrin that we documented in FTC133 cells cultivated on gelatin could therefore play similar functions in other extracellular matrix environments depending on the integrin dimers specifically present at tumor cell surface. The molecular link between LRP-1, calpain activity and β1 integrin stability at cell surface of migrating thyroid carcinoma cells that we established in this study shed new light on the inner control of integrin activity by LRP-1. It also confirms that calpain activity may exert a strong influence on inside-out activation of integrins by controlling the net balance of pro and anti-adhesive signals that determines the adoption of an efficient migrating strategy for invading tumor cells.

An abundant literature described a pro-motile action of calpains linked to the release of tensions exerted at cell-matrix adhesion sites through partial proteolysis of focal contacts and cytoskeleton components (41, 48, 51). Though, a growing number of evidence conversely showed that calpains substrates cleavage can produce or unmask pro-adhesive motifs and stabilize peripheral focal adhesions. Accordingly, calpain-dependent proteolysis of paxillin negatively regulated focal adhesion dynamics and impaired cell migration (52). The binding of talin amino-terminal head domain to the intracellular tail of integrin constitutes a paradigm of inside-out activation of multiple classes of integrins that reinforces their affinity for extracellular ligands (53, 54). Although calpains can reduce the rate of talin stability in adhesion complexes, the local release of its head domain was associated to integrin activation and focal complexes stabilization (55, 56). Ultra-resolutive studies of talin orientation in adhesion sites revealed that its amino-terminal part was in close association with the core focal adhesion area, containing intracellular domains of integrins, whereas its rod carboxy-terminal part was more distal and oriented towards the zone of junction with actin stress fibers (57). We observed that the cell-surface accumulation of β1 integrin upon LRP-1 silencing was partially reversed by calpain inhibition and that the occurrence of the cleaved form of talin appeared to follow the presence of this integrin at the membrane of thyroid carcinoma cells. We previously showed that LRP-1 inhibition resulted in a depletion of talin-enriched complexes from FAK, which could function as a platform for calpain-2 targeting and activation (9, 58). Unlike calpain-2, which was accumulated in LRP-1-silenced cells, calpain-1 expression was surprisingly unaffected. We thus postulate that the increase in calpain activity documented here mainly involves calpain-2 isoform and that LRP-1 is controlling this dominant calpain signal to limit the adhesive strength of FTC133 cells. Capn1 and capn2 genes being located on different chromosomes, and regulated by distinct promoter regions, LRP-1-dependent signals likely mobilize a set of transcription regulators leading to the specific downregulation of calpain-2 transcriptional expression. Of note, most of the reports illustrating pro-adhesive functions of calpains were focused on calpain-2, which is in accordance with our data (41, 48, 59). Following LRP-1 repression, we showed that increased calpain activity coincided with an overspread morphology of tumor cells that accumulated more peripheral talin-positive complexes. This strongly argues for a major role of calpain-2 in favor of focal adhesion maturation and stabilization of integrin-based adhesions. In that context, the maintenance of baseline calpain activity by LRP-1 is therefore permissive to efficient tumor cell motility.

The regulation of calpain activity is complex dynamic and still lacking in mechanistic insights, especially in the field of cancer (41, 49, 60, 61). In this study, we evidenced that calpain activity are dampened by LRP-1 in thyroid carcinoma cells. An in-depth mechanical investigation revealed that this attenuation was in part linked to a specific control of calpain-2 transcription. We tested the potential contribution of two LRP-1-dependent intracellular signals, namely ERK and JNK kinases (28), but we did not evidence any variation of capn2 transcription level in the presence of specific MAPK inhibitors (data not shown). Further studies should thus address the specific LRP-1-dependent signals involved in capn2 gene expression control. In our experiments, the expression level of calpastatin was not affected by LRP-1 expression. MAPK can also influence calpains at post-translational level. Indeed, JNK kinases can phosphorylate some focal adhesion components and calpains substrates with various consequences on cell motility, but also potentially on calpains targeting to adhesion sites (62, 63). Phosphorylation by ERK1/2 MAPK had been directly involved in the activation of calpain 2 (43). We had previously shown that LRP-1 controls both the activation and the incorporation of JNK and ERK kinases to talin-rich focal adhesions (28). We therefore tested a putative cross-reaction of MAPK with calpains by measuring their activities in the presence of specific inhibitors but we could not detect any significant contribution of MAPK to LRP-1-dependent calpains regulation (data not shown). The phosphorylation of S369 residue of calpain-2 by protein kinase A (PKA) was involved in the inhibition of the protease, that subsequently became frozen in an inactive conformation (46). Ligand binding to LRP-1 had previously been involved in the activation of PKA (25). We demonstrated that PKA activity was decreased upon LRP-1 repression in thyroid carcinoma cells. Moreover, specific inhibitors of PKA significantly impaired calpain activity developed upon LRP-1 silencing and we could show by coimmunoprecipitation that the level of serine phosphorylation of calpain-2 was decreased by the PKA inhibitor H-89, specifically in LRP-1 expressing carcinoma cells. These data strongly argue that PKA activation by LRP-1 contributes to the limitation of calpain activity and establish a new functional link between LRP-1, extracellular matrix attachment and invasive properties of carcinoma cells. Among other putative mechanisms that might be at play to control the activity of calpains, are the expression, activity and distribution of calcium channels, which have not tested to date. Although, TRPM4 and PIEZO1 for instance emerge as mechanosensitive regulators of spatio-temporal confinement of calpain activity in various physio-pathological contexts (61, 64, 65). These targets provide an appealing outlook for investigating and completing our view of the multifaceted contributions of LRP-1 to extracellular stimuli and mechanical tensions integration in both nascent and mature integrin-based attachment to various extracellular matrix environments.

Considering the complexity and the multiplicity of the proteolytic targets of calpains, disentangling its control mode of tumor cell migration could be puzzling, even limiting the analysis to integrins adhesome. The level of maturation and clustering of adhesion complexes affects the spatial control of calpain activity and their targets ratio, making the net balance of their proteolytic action difficult to predict. It is interesting to note that similarly to LRP-1 (4, 9), the mobilization of calpains to adhesion sites can be coupled to membrane focalized matrix proteases, such as MT1-MMP, directed in an endocytic/recycling pathway to support persistent migration of tumor cells (47). It is tempting to propose a model where LRP-1 could organize both extracellular and intracellular proteolytic events to control tensional stress accumulating in cell-matrix adhesion sites. Moreover, common intracellular scaffolds shared between LRP-1 and some integrins could result in synergistic or competitive titrations depending on the availability and access of calpains to their substrates. Accordingly, the deregulation of some specific targets of calpains can trigger cancer cell motility and promote their aggressive behavior by displacing calpain activity from anti- to pro-metastatic substrates (66). Our work and concomitant studies from other teams demonstrated the critical role played by LRP-1 in cell polarization based on rear/front partition of focal contacts components, such as FAK or paxillin (9), or actin cytoskeleton regulators Rho and Rac small GTPases (30). Here we showed that calpain inhibition in LRP-1-silenced cells restored the mesenchymal morphology of spread FTC133 cells, suggesting that baseline calpain activity maintained through LRP-1-mediated signals contributed to polarize tumor cells. Ezrin, a cytoskeleton protein incorporated in focal complexes, was involved in the recruitment and the distribution of calpain at membrane protrusion or retraction sites (60). Moreover, the spatial restriction of active calpains can stabilize the formation of nascent filopodiae (67) as well as the maturation of dual adhesive and invasive structures such as invadopodiae (59), which could predispose cancer cells to aggressive behavior *in vivo*. A recent *in vivo* study described that calpains constitute crucial determinants of tumor cell dissemination strategies. Indeed, under hypoxic conditions, induction of calpain-2 switched the collective mode of migration of breast and head and neck cancer cells to an amoeboid metastatic behavior. This transition relied on talin cleavage and decreased strength of β1 integrin-mediated attachments, and was reverted by pharmacological challenge of calpain activity (49).

Like most drug targetable molecules that are deregulated during cancer progression, a major clinical challenge in practice could rely on the highly specific and contextual cleavage products of calpains and their associated metabolic consequences on cancer cells as well as the reactive and adaptive tumor bed. However, the novel LRP-1-mediated pathway that we identified enrich the panel of versatile functions exerted by the receptor, once again detected at the crossroad between extracellular cues and membrane dynamics, and further illustrates its crucial role in cell-matrix adhesions adaptation to the microenvironment of cancer cells.

## Data availability statement

The raw data supporting the conclusions of this article will be made available by the authors, without undue reservation.

## Author contributions

BL and SD designed the study. BL, JM, CS, CH, CT, DR, LM, LT, SS and SD collected, interpreted, and analyzed the data. BL wrote the manuscript. All the authors revised the manuscript critically for important intellectual content and read and approved the final manuscript.

## Funding

The project leading to this publication has received funding from Université de Reims Champagne-Ardenne, CNRS and Ligue contre le Cancer.

## Acknowledgments

We thank Mrs Arlette Thomachot for her valuable editing assistance.

## Conflict of interest

SD is co-founder of Apmonia-Therapeutics and acts as the chair of the Scientific and Clinical Advisory Board.

The remaining authors declare that the research was conducted in the absence of any commercial or financial relationships that could be construed as a potential conflict of interest.

## Publisher’s note

All claims expressed in this article are solely those of the authors and do not necessarily represent those of their affiliated organizations, or those of the publisher, the editors and the reviewers. Any product that may be evaluated in this article, or claim that may be made by its manufacturer, is not guaranteed or endorsed by the publisher.

## Abbreviations

LRP-1, low-density lipoprotein receptor-related protein-1; PKA, protein kinase A; MMP, matrix metalloprotease; uPA, urokinase-type plasminogen activator; tPA, tissue-type plasminogen activator; JAK/Stat, janus kinase/signal transducer and activator of transcription; PAI-1, plasminogen activator inhibitor 1; ERK, extracellular signal-regulated kinase; HSP, heat shock protein; FTC, follicular thyroid carcinoma; ECM, extracellular matrix; LFA-1, lymphocyte function-associated antigen-1; FAK, focal adhesion kinase; JNK, c-Jun N-terminal kinase; MAPK, mitogen-activated protein kinase; TRPM4, transient receptor potential cation channel subfamily M; PIEZO1, piezo-type mechanosensitive ion channel component 1; MT1-MMP, membrane type 1 matrix metalloprotease; CAPN2, calpain-2; FA, focal adhesion.

## References

[1] Herz J Strickland DK. LRP: A multifunctional scavenger and signaling receptor. J Clin Invest (2001) 108(6):779–84. doi: 10.1172/JCI200113992 PMC200939 11560943

[2] Strickland DK Ashcom JD Williams S Burgess WH Migliorini M Argraves WS. Sequence identity between the alpha 2-macroglobulin receptor and low density lipoprotein receptor-related protein suggests that this molecule is a multifunctional receptor. J Biol Chem (1990) 265(29):17401–4. doi: 10.1016/S0021-9258(18)38172-9 1698775

[3] Lillis AP Van Duyn LB Murphy-Ullrich JE Strickland DK. LDL receptor-related protein 1: unique tissue-specific functions revealed by selective gene knockout studies. Physiol Rev (2008) 88(3):887–918. doi: 10.1152/physrev.00033.2007 18626063 PMC2744109

[4] Van Gool B Dedieu S Emonard H Roebroek AJ. The matricellular receptor lrp1 forms an interface for signaling and endocytosis in modulation of the extracellular tumor environment. Front Pharmacol (2015) 6:271. doi: 10.3389/fphar.2015.00271 26617523 PMC4639618

[5] van der Geer P. Phosphorylation of LRP1: regulation of transport and signal transduction. Trends Cardiovasc Med (2002) 12(4):160–5. doi: 10.1016/S1050-1738(02)00154-8 12069755

[6] Bell RD Deane R Chow N Long X Sagare A Singh I. SRF and myocardin regulate LRP-mediated amyloid-beta clearance in brain vascular cells. Nat Cell Biol (2009) 11(2):143–53. doi: 10.1038/ncb1819 PMC2654279 19098903

[7] Boucher P Gotthardt M Li WP Anderson RG Herz J. LRP: role in vascular wall integrity and protection from atherosclerosis. Science (2003) 300(5617):329–32. doi: 10.1126/science.1082095 12690199

[8] Hu K Wu C Mars WM Liu Y. Tissue-type plasminogen activator promotes murine myofibroblast activation through LDL receptor-related protein 1-mediated integrin signaling. J Clin Invest (2007) 117(12):3821–32. doi: 10.1172/JCI32301 PMC2082143 18037995

[9] Dedieu S Langlois B Devy J Sid B Henriet P Sartelet H. LRP-1 silencing prevents malignant cell invasion despite increased pericellular proteolytic activities. Mol Cell Biol (2008) 28(9):2980–95. doi: 10.1128/MCB.02238-07 PMC2293087 18316405

[10] Cao C Lawrence DA Li Y Von Arnim CA Herz J Su EJ. Endocytic receptor LRP together with tPA and PAI-1 coordinates mac-1-dependent macrophage migration. EMBO J (2006) 25(9):1860–70. doi: 10.1038/sj.emboj.7601082 PMC1456942 16601674

[11] Sid B Dedieu S Delorme N Sartelet H Rath GM Bellon G. Human thyroid carcinoma cell invasion is controlled by the low density lipoprotein receptor-related protein-mediated clearance of urokinase plasminogen activator. Int J Biochem Cell Biol (2006) 38(10):1729–40. doi: 10.1016/j.biocel.2006.04.005 16807059

[12] Etique N Verzeaux L Dedieu S Emonard H. LRP-1: A checkpoint for the extracellular matrix proteolysis. BioMed Res Int (2013) 2013:152163. doi: 10.1155/2013/152163 23936774 PMC3723059

[13] Amos S Mut M diPierro CG Carpenter JE Xiao A Kohutek ZA. Protein kinase c-alpha-mediated regulation of low-density lipoprotein receptor related protein and urokinase increases astrocytoma invasion. Cancer Res (2007) 67(21):10241–51. doi: 10.1158/0008-5472.CAN-07-0030 PMC2386949 17974965

[14] Kancha RK Stearns ME Hussain MM. Decreased expression of the low density lipoprotein receptor-related protein/alpha 2-macroglobulin receptor in invasive cell clones derived from human prostate and breast tumor cells. Oncol Res (1994) 6(8):365–72. 7534510

[15] Fayard B Bianchi F Dey J Moreno E Djaffer S Hynes NE. The serine protease inhibitor protease nexin-1 controls mammary cancer metastasis through LRP-1-mediated MMP-9 expression. Cancer Res (2009) 69(14):5690–8. doi: 10.1158/0008-5472.CAN-08-4573 19584287

[16] Montel V Gaultier A Lester RD Campana WM Gonias SL. The low-density lipoprotein receptor-related protein regulates cancer cell survival and metastasis development. Cancer Res (2007) 67(20):9817–24. doi: 10.1158/0008-5472.CAN-07-0683 17942912

[17] Gaultier A Salicioni AM Arandjelovic S Gonias SL. Regulation of the composition of the extracellular matrix by low density lipoprotein receptor-related protein-1: Activities based on regulation of mRNA expression. J Biol Chem (2006) 281(11):7332–40. doi: 10.1074/jbc.M511857200 16407289

[18] Hu K Yang J Tanaka S Gonias SL Mars WM Liu Y. Tissue-type plasminogen activator acts as a cytokine that triggers intracellular signal transduction and induces matrix metalloproteinase-9 gene expression. J Biol Chem (2006) 281(4):2120–7. doi: 10.1074/jbc.M504988200 16303771

[19] Song H Li Y Lee J Schwartz AL Bu G. Low-density lipoprotein receptor-related protein 1 promotes cancer cell migration and invasion by inducing the expression of matrix metalloproteinases 2 and 9. Cancer Res (2009) 69(3):879–86. doi: 10.1158/0008-5472.CAN-08-3379 PMC2633434 19176371

[20] Gotthardt M Trommsdorff M Nevitt MF Shelton J Richardson JA Stockinger W. Interactions of the low density lipoprotein receptor gene family with cytosolic adaptor and scaffold proteins suggest diverse biological functions in cellular communication and signal transduction. J Biol Chem (2000) 275(33):25616–24. doi: 10.1074/jbc.M000955200 10827173

[21] Guttman M Betts GN Barnes H Ghassemian M van der Geer P Komives EA. Interactions of the NPXY microdomains of the low density lipoprotein receptor-related protein 1. Proteomics (2009) 9(22):5016–28. doi: 10.1002/pmic.200900457 PMC2862490 19771558

[22] Hu K Lin L Tan X Yang J Bu G Mars WM. tPA protects renal interstitial fibroblasts and myofibroblasts from apoptosis. J Am Soc Nephrol (2008) 19(3):503–14. doi: 10.1681/ASN.2007030300 PMC2391054 18199803

[23] Yang M Huang H Li J Li D Wang H. Tyrosine phosphorylation of the LDL receptor-related protein (LRP) and activation of the ERK pathway are required for connective tissue growth factor to potentiate myofibroblast differentiation. FASEB J (2004) 18(15):1920–1. doi: 10.1096/fj.04-2357fje 15469966

[24] Zhang H Lee JM Wang Y Dong L Ko KW Pelletier L. Mutational analysis of the FXNPXY motif within LDL receptor-related protein 1 (LRP1) reveals the functional importance of the tyrosine residues in cell growth regulation and signal transduction. Biochem J (2008) 409(1):53–64. doi: 10.1042/BJ20071127 17908054

[25] Zhu Y Hui DY. Apolipoprotein e binding to low density lipoprotein receptor-related protein-1 inhibits cell migration *via* activation of cAMP-dependent protein kinase a. J Biol Chem (2003) 278(38):36257–63. doi: 10.1074/jbc.M303171200 12857755

[26] Degryse B Neels JG Czekay RP Aertgeerts K Kamikubo Y Loskutoff DJ. The low density lipoprotein receptor-related protein is a motogenic receptor for plasminogen activator inhibitor-1. J Biol Chem (2004) 279(21):22595–604. doi: 10.1074/jbc.M313004200 15001579

[27] Webb DJ Nguyen DH Gonias SL. Extracellular signal-regulated kinase functions in the urokinase receptor-dependent pathway by which neutralization of low density lipoprotein receptor-related protein promotes fibrosarcoma cell migration and matrigel invasion. J Cell Sci (2000) 113(Pt 1):123–34. doi: 10.1242/jcs.113.1.123 10591631

[28] Langlois B Perrot G Schneider C Henriet P Emonard H Martiny L. LRP-1 promotes cancer cell invasion by supporting ERK and inhibiting JNK signaling pathways. PloS One (2010) 5(7):e11584. doi: 10.1371/journal.pone.0011584 20644732 PMC2904376

[29] Cheng CF Fan J Fedesco M Guan S Li Y Bandyopadhyay B. Transforming growth factor alpha (TGFalpha)-stimulated secretion of HSP90alpha: using the receptor LRP-1/CD91 to promote human skin cell migration against a TGFbeta-rich environment during wound healing. Mol Cell Biol (2008) 28(10):3344–58. doi: 10.1128/MCB.01287-07 PMC2423165 18332123

[30] Mantuano E Inoue G Li X Takahashi K Gaultier A Gonias SL. The hemopexin domain of matrix metalloproteinase-9 activates cell signaling and promotes migration of schwann cells by binding to low-density lipoprotein receptor-related protein. J Neurosci (2008) 28(45):11571–82. doi: 10.1523/JNEUROSCI.3053-08.2008 PMC3837707 18987193

[31] Orr AW Pallero MA Murphy-Ullrich JE. Thrombospondin stimulates focal adhesion disassembly through gi- and phosphoinositide 3-kinase-dependent ERK activation. J Biol Chem (2002) 277(23):20453–60. doi: 10.1074/jbc.M112091200 11923291

[32] Song H Bu G. MicroRNA-205 inhibits tumor cell migration through down-regulating the expression of the LDL receptor-related protein 1. Biochem Biophys Res Commun (2009) 388(2):400–5. doi: 10.1016/j.bbrc.2009.08.020 PMC2741500 19665999

[33] Tian Y Wang C Chen S Liu J Fu Y Luo Y. Extracellular Hsp90alpha and clusterin synergistically promote breast cancer epithelial-to-mesenchymal transition and metastasis *via* LRP1. J Cell Sci (2019) 132(15). doi: 10.1242/jcs.228213 31273033

[34] Xue N Du T Lai F Jin J Ji M Chen X. Secreted HSP90alpha-LRP1 signaling promotes tumor metastasis and chemoresistance in pancreatic cancer. Int J Mol Sci (2022) 23(10). doi: 10.3390/ijms23105532 PMC9141888 35628341

[35] Dedieu S Langlois B. LRP-1: A new modulator of cytoskeleton dynamics and adhesive complex turnover in cancer cells. Cell Adh Migr (2008) 2(2):77–80. doi: 10.4161/cam.2.2.6374 19271352 PMC2634989

[36] Perrot G Langlois B Devy J Jeanne A Verzeaux L Almagro S. LRP-1–CD44, a new cell surface complex regulating tumor cell adhesion. Mol Cell Biol (2012) 32(16):3293–307. doi: 10.1128/MCB.00228-12 PMC3434541 22711991

[37] Spijkers PP da Costa Martins P Westein E Gahmberg CG Zwaginga JJ Lenting PJ. LDL-receptor-related protein regulates beta2-integrin-mediated leukocyte adhesion. Blood (2005) 105(1):170–7. doi: 10.1182/blood-2004-02-0498 15328156

[38] Zemskov EA Mikhailenko I Strickland DK Belkin AM. Cell-surface transglutaminase undergoes internalization and lysosomal degradation: An essential role for LRP1. J Cell Sci (2007) 120(Pt 18):3188–99. doi: 10.1242/jcs.010397 17711877

[39] Theret L Jeanne A Langlois B Hachet C David M Khrestchatisky M. Identification of LRP-1 as an endocytosis and recycling receptor for beta1-integrin in thyroid cancer cells. Oncotarget (2017) 8(45):78614–32. doi: 10.18632/oncotarget.20201 PMC5667986 29108253

[40] Wujak L Bottcher RT Pak O Frey H El Agha E Chen Y. Low density lipoprotein receptor-related protein 1 couples beta1 integrin activation to degradation. Cell Mol Life Sci (2018) 75(9):1671–85. doi: 10.1007/s00018-017-2707-6 PMC11105666 29116364

[41] Franco SJ Huttenlocher A. Regulating cell migration: calpains make the cut. J Cell Sci (2005) 118(Pt 17):3829–38. doi: 10.1242/jcs.02562 16129881

[42] Su LT Agapito MA Li M Simonson WT Huttenlocher A Habas R. TRPM7 regulates cell adhesion by controlling the calcium-dependent protease calpain. J Biol Chem (2006) 281(16):11260–70. doi: 10.1074/jbc.M512885200 PMC3225339 16436382

[43] Glading A Bodnar RJ Reynolds IJ Shiraha H Satish L Potter DA. Epidermal growth factor activates m-calpain (calpain II), at least in part, by extracellular signal-regulated kinase-mediated phosphorylation. Mol Cell Biol (2004) 24(6):2499–512. doi: 10.1128/MCB.24.6.2499-2512.2004 PMC355832 14993287

[44] Sid B Langlois B Sartelet H Bellon G Dedieu S Martiny L. Thrombospondin-1 enhances human thyroid carcinoma cell invasion through urokinase activity. Int J Biochem Cell Biol (2008) 40(9):1890–900. doi: 10.1016/j.biocel.2008.01.023 18321763

[45] Goette A Arndt M Rocken C Staack T Bechtloff R Reinhold D. Calpains and cytokines in fibrillating human atria. Am J Physiol Heart Circ Physiol (2002) 283(1):H264–72. doi: 10.1152/ajpheart.00505.2001 12063299

[46] Shiraha H Glading A Chou J Jia Z Wells A. Activation of m-calpain (calpain II) by epidermal growth factor is limited by protein kinase a phosphorylation of m-calpain. Mol Cell Biol (2002) 22(8):2716–27. doi: 10.1128/MCB.22.8.2716-2727.2002 PMC133710 11909964

[47] Bravo-Cordero JJ Cordani M Soriano SF Diez B Munoz-Agudo C Casanova-Acebes M. A novel high-content analysis tool reveals Rab8-driven cytoskeletal reorganization through rho GTPases, calpain and MT1-MMP. J Cell Sci (2016) 129(8):1734–49. doi: 10.1242/jcs.174920 26940916

[48] Bate N Gingras AR Bachir A Horwitz R Ye F Patel B. Talin contains a c-terminal calpain2 cleavage site important in focal adhesion dynamics. PloS One (2012) 7(4):e34461. doi: 10.1371/journal.pone.0034461 22496808 PMC3319578

[49] Te Boekhorst V Jiang L Mahlen M Meerlo M Dunkel G Durst FC. Calpain-2 regulates hypoxia/HIF-induced plasticity toward amoeboid cancer cell migration and metastasis. Curr Biol (2022) 32(2):412–27 e8. doi: 10.1016/j.cub.2021.11.040 34883047 PMC10439789

[50] Zhao Y Malinin NL Meller J Ma Y West XZ Bledzka K. Regulation of cell adhesion and migration by kindlin-3 cleavage by calpain. J Biol Chem (2012) 287(47):40012–20. doi: 10.1074/jbc.M112.380469 PMC3501071 23012377

[51] Calle Y Carragher NO Thrasher AJ Jones GE. Inhibition of calpain stabilises podosomes and impairs dendritic cell motility. J Cell Sci (2006) 119(Pt 11):2375–85. doi: 10.1242/jcs.02939 16723743

[52] Cortesio CL Boateng LR Piazza TM Bennin DA Huttenlocher A. Calpain-mediated proteolysis of paxillin negatively regulates focal adhesion dynamics and cell migration. J Biol Chem (2011) 286(12):9998–10006. doi: 10.1074/jbc.M110.187294 21270128 PMC3060554

[53] Tadokoro S Shattil SJ Eto K Tai V Liddington RC de Pereda JM. Talin binding to integrin beta tails: A final common step in integrin activation. Science (2003) 302(5642):103–6. doi: 10.1126/science.1086652 14526080

[54] Wegener KL Partridge AW Han J Pickford AR Liddington RC Ginsberg MH. Structural basis of integrin activation by talin. Cell (2007) 128(1):171–82. doi: 10.1016/j.cell.2006.10.048 17218263

[55] Yan B Calderwood DA Yaspan B Ginsberg MH. Calpain cleavage promotes talin binding to the beta 3 integrin cytoplasmic domain. J Biol Chem (2001) 276(30):28164–70. doi: 10.1074/jbc.M104161200 11382782

[56] Huang C Rajfur Z Yousefi N Chen Z Jacobson K Ginsberg MH. Talin phosphorylation by Cdk5 regulates Smurf1-mediated talin head ubiquitylation and cell migration. Nat Cell Biol (2009) 11(5):624–30. doi: 10.1038/ncb1868 PMC2714540 19363486

[57] Liu J Wang Y Goh WI Goh H Baird MA Ruehland S. Talin determines the nanoscale architecture of focal adhesions. Proc Natl Acad Sci U S A. (2015) 112(35):E4864–73. doi: 10.1073/pnas.1512025112 PMC4568271 26283369

[58] Carragher NO Westhoff MA Fincham VJ Schaller MD Frame MC. A novel role for FAK as a protease-targeting adaptor protein: regulation by p42 ERK and src. Curr Biol (2003) 13(16):1442–50. doi: 10.1016/S0960-9822(03)00544-X 12932330

[59] Cortesio CL Chan KT Perrin BJ Burton NO Zhang S Zhang ZY. Calpain 2 and PTP1B function in a novel pathway with src to regulate invadopodia dynamics and breast cancer cell invasion. J Cell Biol (2008) 180(5):957–71. doi: 10.1083/jcb.200708048 PMC2265405 18332219

[60] Hoskin V Szeto A Ghaffari A Greer PA Cote GP Elliott BE. Ezrin regulates focal adhesion and invadopodia dynamics by altering calpain activity to promote breast cancer cell invasion. Mol Biol Cell (2015) 26(19):3464–79. doi: 10.1091/mbc.E14-12-1584 PMC4591691 26246600

[61] McHugh BJ Murdoch A Haslett C Sethi T. Loss of the integrin-activating transmembrane protein Fam38A (Piezo1) promotes a switch to a reduced integrin-dependent mode of cell migration. PloS One (2012) 7(7):e40346. doi: 10.1371/journal.pone.0040346 22792288 PMC3390408

[62] Huang C Rajfur Z Borchers C Schaller MD Jacobson K. JNK phosphorylates paxillin and regulates cell migration. Nature (2003) 424(6945):219–23. doi: 10.1038/nature01745 12853963

[63] Smadja-Lamere N Boulanger MC Champagne C Branton PE Lavoie JN. JNK-mediated phosphorylation of paxillin in adhesion assembly and tension-induced cell death by the adenovirus death factor E4orf4. J Biol Chem (2008) 283(49):34352–64. doi: 10.1074/jbc.M803364200 PMC2662241 18818208

[64] Saxena M Changede R Hone J Wolfenson H Sheetz MP. Force-induced calpain cleavage of talin is critical for growth, adhesion development, and rigidity sensing. Nano Lett (2017) 17(12):7242–51. doi: 10.1021/acs.nanolett.7b02476 PMC7490970 29052994

[65] Aglialoro F Hofsink N Hofman M Brandhorst N van den Akker E. Inside out integrin activation mediated by piezo1 signaling in erythroblasts. Front Physiol (2020) 11:958. doi: 10.3389/fphys.2020.00958 32848880 PMC7411472

[66] Xu Y Bismar TA Su J Xu B Kristiansen G Varga Z. Filamin a regulates focal adhesion disassembly and suppresses breast cancer cell migration and invasion. J Exp Med (2010) 207(11):2421–37. doi: 10.1084/jem.20100433 PMC2964581 20937704

[67] Jacquemet G Baghirov H Georgiadou M Sihto H Peuhu E Cettour-Janet P. L-type calcium channels regulate filopodia stability and cancer cell invasion downstream of integrin signalling. Nat Commun (2016) 7:13297. doi: 10.1038/ncomms13297 27910855 PMC5146291

